# Potential biological efficacy of Pinus plant species against oxidative, inflammatory and microbial disorders

**DOI:** 10.1186/s12906-016-1011-6

**Published:** 2016-01-28

**Authors:** Aditi Sharma, Rohit Goyal, Lalit Sharma

**Affiliations:** 1 School of Pharmaceutical Sciences, Shoolini University, Solan, Himachal Pradesh India; 2 Jaypee University of Information Technology, Waknaghat, Solan, Himachal Pradesh India

**Keywords:** Pinus, Antioxidant, Ant-inflammatory, Antibacterial and Antifungal

## Abstract

**Background:**

Traditionally, Pine has been used to treat oxidative and inflammatory disorders. The study was aimed to investigate biological potential of phytoconstituents of Pinus plant species: *Pinus roxburghii*, *Pinus wallichiana* and *Pinus gerardiana* using *in-vitro* antioxidant, anti-inflammatory and antimicrobial methods.

**Method:**

The hydro-alcoholic extraction of dried plant: stem bark was done and the antioxidant activity was evaluated using free radical scavenging methods for 1,1-diphenyl-2-picrylhydrazyl, (DPPH), nitric oxide and hydrogen peroxide radicals, reducing power assays, and total antioxidant capacity. Anti-inflammatory activity was carried out using albumin denaturation and HRBC membrane stabilization assays. Antimicrobial and antifungal activities were also conducted using agar well diffusion method.

**Results:**

The qualitative phytochemical analysis of hydro-alcoholic stem bark extracts of three plant species revealed the presence of various biochemical compounds such as alkaloids, flavonoids, glycosides, triterpenoids and saponins. Quantitative phytochemical analysis of plant extracts showed the presence of phenolics, flavonoids, tannins, beta-carotene and lycopene. Plant extracts of three pinus species showed significant antioxidant activity against DPPH, nitric oxide and H_2_O_2_ radicals. In *in-vitro* anti‐inflammatory investigation, *Pinus roxburghii* exhibited highest protection against albumin denaturation 86.54 ± 1.85 whereas *Pinus gerardiana* showed 82.03 ± 2.67. Moreover, plant extracts were found to prevent the growth of microorganisms *Pseudomonas aeruginosa, Escherichia coli*, *Staphylococcus aureus* and *Candida albicans* showing promising antibacterial and antifungal activities against*Candida albicans*.

**Conclusion:**

The findings of the present study derived the rational for the therapeutic usage of Pinus which is a highly timber yielding plant from Himalayan region, against oxidative, inflammatory and microbial diseases.

## Background

The chronic pathological conditions like atherosclerosis, cancer, diabetes, rheumatoid arthritis, Alzheimer’s disease, myocardial infarction are recognized majorly with over production of free radicals, which cause oxidative damage to biomolecules: lipids, proteins and DNA and imbalance between generation of reactive oxygen species (ROS) and antioxidant defense system [1, 2]. The free radicals are mainly inclusive of ROS like superoxide anion (O_2_·), hydroxyl (OH·), hydroperoxyl (OOH·), peroxyl (ROO·) radicals and reactive nitrogen species (RNS) like nitric oxide (NO·), peroxynitrite (ONOO·) and nitrogen dioxide (NO_2_) radicals [3, 4]. Increased production of free radicals and oxidative damage result in an inflammatory pathological state characterized by increased expression of pro-inflammatory mediators, cytokines, chemokines *i.e.* TNF-alpha, interleukins, recruitment of adhesion molecules and caspases [5–7]. This oxidative and inflammatory state is prone for the occurrence of microbial infection due to the presence of microorganisms. Bacterial infections of CNS involve acute bacterial meningitis caused by *Haemophillus influenzae, Neisseria. meningitidis, Streptococcus pneumoniae* or streptococcus microorganisms leading to epilepsy, learning deficits and other neurological disabilities. Antioxidants are considered possible protection for human body that reduces oxidative damage by scavenging these free radicals.

The Indian Himalayan region, a birthplace of Ayurveda and alternative therapies, covers about 18 % of India and extends more than 2,800 km long and 220–300 km wide with altitudes of 200–8000 m and fulfils a very large proportion of medicinal plants from 80 % of Ayurveda medicine, 46 % of Unani drugs and 33 % of allopathic drugs developed from India [8–10]. WHO estimates that 80 % of earth inhabitants rely on traditional medicine. The unique climatic conditions enable a rich array of growth of various medicinally useful plants [10]. Pinus species are important forest primarily for timber interests and source of gum oleoresins. Three species of Pinus plants are abundantly found *i.e. Pinus roxburghii, Pinus wallichiiana* and *Pinus gerardiana* which belong to the family: Pinaceae. *P. roxburghii* Sarg, commonly called as Chir pine, is a tall tree with spreading crown, at altitude 450–2400 m from Kashmir to Bhutan and Siwalik hills [11]. *P. wallichiana* also known as blue pine, found at an altitude 2000–3500 m whereas *P. gerardiana*, commonly called as Chilgoza which are found at an altitude of 1600–3000 m in Kinnaur district of Himachal Pradesh (HP). *P. roxburghii* is reported to possess analgesic, anti-inflammatory, hepatoprotective, antibacterial, anticonvulsant and anti-dyslipidemic activities. It is being used locally as charcoal, pigment, herbicide, and for resin and wood [12–16].

According to Ayurvedic methodology, the vitiated state of special elements: vata, pitta and kapha doshas result body sickness which is attributed to increased production of free radicals, inflammatory enzymes and altered immune response [17]. The constituents of Pine (essential oil) reduces surplus of vata and kapha and treat pitta deficiency. In Ayurveda, Pinus species are recommended to cure jvara (fever) and svedadaurgandhya (foul smell because of excessive sweating) [18]. Phytochemically, it is reported to contain constituents like terpenoids, flavonoids, tannins, and xanthones. The resin is composed of car-3-ene, pinene, longifolene, camphene, limonene, α-terpinene, α-terpineol, d-borneol and dl-camphor [19, 20]. *P. wallichiana* is exploited for timber and used for the production of turpentine oil, rosin, needle oil and camphor [21, 22]. The nuts contain 50 % fat, 30 % protein, 10 % carbohydrate, 4 % ash and 6 % moisture [23, 24]. The detailed phytochemical and biological evaluations of different species of Pine are still to be explored. The recent researches for the search of natural candidate with potent biological activity have been directed to combat with oxidative, inflammatory and microbial reactions. Moreover, there is no report available mentioning the biological potential of various plant components from Pinus. Therefore, the present study was aimed to investigate antioxidant, anti-inflammatory and antimicrobial effects *in-vitro* of extracts of three Pinus plant species: *P. roxburghii*, *P. wallichiana* and *P. gerardiana*.

## Methods

### Collection of plant material

Stem bark of *P.roxburghii* was collected from local areas of Solan, HP, bark of *P. wallichiana* collected from Shimla, HP and bark of *P.gerardiana* collected from Rekongpeo, Kinnaur, HP. All plant drug samples were duly authenticated from Department of Forestry, YS Parmar University of Horticulture and Agriculture Sciences, Nauni, HP, India and samples were kept in institutional herbarium with voucher specimen Nos.13488, 13489, 13506. The plant part was dried in shade, powdered by the mechanical grinder and stored in air tight container till further use.

### Preparation of extracts

The powdered plant material of stem bark was defatted using petroleum ether and extracted with soxhlet apparatus using 90 % *v/v* ethanol in water (hydro-alcoholic extraction). The solvent was recovered by evaporation under reduced pressure using rota evaporator. The semisolid mass was further freeze dried using lyophilizer at -80 °C for 24 h.

### Chemicals

1,1-diphenyl-2-picrylhydrazyl (DPPH), rutin, naphthylethylenediamine dichloride, and standard markers for HPLC Gallic acid, tannic acid and quercetin were purchased from Sigma Chemicals. Ferric chloride, vanillin, trichloroacetic acid (TCA), Folin-Ciocalteu’s reagent, aluminium chloride (AlCl3) were purchased from Himedia Pvt Ltd. All other chemicals used in the present study were of analytical grade.

### Phytochemical screening of plant extracts

The prepared hydroalcoholic extracts of all three plants were subjected to phytochemical screening tests to evaluate the presence of chemical constituents. The extracts was treated with Mayer’s reagent (Potassium mercuric iodide: formation of yellow coloured precipitate); Wagner’s reagent (Iodine in potassium iodide: formation of red brown/reddish precipitate); Dragendroff’s reagent (solution of potassium bismuth iodide: formation of red precipitate indicated the presence of alkaloids. The extract was boiled with 0.25 % *w/v* ninhydrin reagent; formation of blue colour indicated the presence of amino acids and proteins. A blackish red colour resulting from the addition of ferric chloride reagent to extracts filtrate indicated the presence of flavonoids. Occurrence of violet ring at the junction when extracts filtrate was treated with 2 drops of alcoholic α-naphthol solution was indicative of carbohydrates (Mollisch test). Fats and oils were detected with Sudan 3 treatment. 1 % gelatin solution containing sodium chloride was added to the extract, white precipitate showed the presence of tannins. Test solution was mixed with water and shaken; the formation of 1 cm froth was an indication of saponin glycoside. Salkowaski, sulphur powder test was done for steroids. Terpenoids were detected by formation of yellow precipitate when treated with lead acetate [25, 26].

### Determination of total flavonoid content

Total flavonoid content (TFC) was determined by aluminium chloride assay using calorimetric estimation [27]. In different test tubes, 0.5 ml extract, 2 ml of distilled water, followed by 0.15 ml of sodium nitrite (5 % *w/v*) was added. After 5 min, 0.15 ml of aluminium trichloride (10 %) was added and incubated for 6 min. After incubation 2 ml of sodium hydroxide (4 % *w/v*) was added. After 15 min of incubation reaction mixture turns to pink and absorbance was measured against blank *e.g.* distilled water at 510 nm. A natural flavonoid rutin was used as standard. The TFC was expressed in mg of rutin equivalents per gram of extract.

### Determination of total phenolic content

The total phenolic content was estimated according to Folin-ciocalteu phenol reagent method [28]. The solution of gallic acid was prepared in 80 % methanol for the standard curve. Folin-ciocalteu reagent was added to 100 μl of sample in ratio 1:10. The solution was mixed and incubated at room temperature for 1 min followed by the addition of 1.5 ml of 20 % sodium carbonate. Final mixture was shaken and incubated for 90 min in the dark at room temperature. The absorbance was taken at 725 nm and the phenolic content was expressed as Gallic acid equivalents GAE/g of sample.

### Condensed tannin quantification

A volume (50 ml) of concentrations (100 mg/ml) of plant extract or standard solution of catechin (CE) was mixed with 3 ml of 4 % vanillin methanol solution. 1.5 ml of concentrated hydrochloric acid was added and 15 min after; the absorbance was measured against blank using distilled water at 510 nm. Tannin content was expressed as mg CE/g of sample, using a catechin calibration curve [29].

### Estimation of β-carotene and lycopene

β- Carotene and Lycopene were determined according to the method of Nagata and Yamashita [30]. The dried extract was vigorously shaken with 10 ml of acetone-hexane mixture (4:6) for 1 min and filtered through Whatman No.4 filter paper. The absorbance of filtrate was measured at 453, 505, 645 and 663 nm. The content of β-carotene and lycopene were calculated using following equations:

$$ \begin{array}{l}\mathrm{Lycopene}\ \left(\mathrm{mg}/100\ \mathrm{ml}\right) = - 0.0458{\mathrm{A}}_{663} + 0.372{\mathrm{A}}_{505} + 0.0806{\mathrm{A}}_{453}\\ {}\upbeta \hbox{-}\ \mathrm{Carotene}\ \left(\mathrm{mg}/100\ \mathrm{ml}\right) = 0.216{\mathrm{A}}_{663} - 0.304{\mathrm{A}}_{505} + 0.452{\mathrm{A}}_{453.}\end{array} $$

The values are expressed as μg/g of extract.

### Evaluation of free radical scavenging activity

#### 1-1 Diphenyl-2-picrylhydrazyl (DPPH) radical scavenging assay

The free radical scavenging activity of prepared samples was determined according to ability of extract to bleach to stable DPPH radicals. 0.5 ml of DPPH was added to 0.5 ml aliquots of standard or test solution in different concentrations: 10, 20, 40, 80, 160, 180, 200 μg/ml. Control test tubes were loaded with 0.5 mL of Dimethyl sulfoxide (DMSO) and 0.5 mL DPPH. After incubation at 37 °C for 30 min in dark, the absorbance was recorded at 517 nm. Ascorbic acid was used as a standard [31, 32]. The percentage scavenging by test sample at each concentration was calculated using following formula:

$$ \mathrm{Scavenging}\ \mathrm{DPPH}\ \left(\%\right) = \left[\left({\mathrm{Abs}}_{\mathrm{control}}\hbox{--} {\mathrm{Abs}}_{\mathrm{sample}}\right)\ /{\mathrm{Abs}}_{\mathrm{control}}\right] \times 100 $$

IC_50_ represents the level where 50 % of radicals scavenged by test or standard sample.

#### Nitric oxide scavenging assay

An inhibition of nitric oxide radicals was estimated using the Griess reaction method. Griess reagent was prepared by mixing 1 % sulphanilamide in 5 % *v/v* phosphoric acid and 0.01 % naphthylethylenediamine in distilled water in equal volumes. The solution of sodium nitroprusside (5 mM) in standard phosphate buffer (0.025 M, pH 7.4) was prepared and incubated with different concentrations of standard and test sample: 10, 20, 40, 80, 160, 180 and 200 μg/ml at 37 °C for 5 h. An equivalent amount of methanol was taken as control. After 5 h, 0.05 ml of incubated solution was removed and diluted with 0.5 ml of Griess reagent. The absorbance of chromophore formed during the digitization of nitrite with sulphanilamide and its subsequent coupling with naphthylethylenediamine was read at 546 nm. Ascorbic acid was used as a standard [33, 34]. The percentage scavenging by test fractions at each concentration was calculated using following formula:

$$ \mathrm{Scavenging}\ \mathrm{NO}\ \left(\%\right) = \left[\left({\mathrm{Abs}}_{\mathrm{control}}-{\mathrm{Abs}}_{\mathrm{sample}}\right)/{\mathrm{Abs}}_{\mathrm{control}}\right] \times 100 $$

IC_50_ represents the level where 50 % of radicals scavenged by test or standard sample.

#### Hydrogen peroxide (H_2_O_2_) scavenging assay

The ability of extract to scavenge H_2_O_2_ was determined according to method of Khaled-Khodjaa [35]. The solution of H_2_O_2_ (40 mM) was prepared in 50 mM phosphate buffer (pH 7.4). Different concentrations of sample and standard, 10, 20, 40, 80, 160, 180, 200 μg/ml (1.2 ml) were added to a H_2_O_2_ solution (0.6 ml). After 10 min, absorbance of H_2_O_2_ at 230 nm was determined against a blank solution containing phosphate buffer, ascorbic acid used as reference compound. The percentage of H_2_O_2_ scavenged by the sample was calculated using following formula:

$$ \mathrm{Scavenged}\ {\mathrm{H}}_2{\mathrm{O}}_2 = {\mathrm{Abs}}_{\mathrm{control}}\hbox{--}\ {\mathrm{Abs}}_{\mathrm{sample}}/{\mathrm{Abs}}_{\mathrm{control}} \times 100 $$

#### Reducing power assay

The reducing power of extracts was determined by the method of Oyaizu [36]. Briefly, 1 ml of sample was mixed with 2.5 ml of phosphate buffer (0.2 M, pH 6.6) and 2.5 ml of potassium Ferricyanide (1 %). The reaction mixture was incubated at 50 °C for 20 min. Then 2.5 ml of trichloroacetic acid (10 %) was added and centrifuged for 10 min. An aliquot 2.5 ml was mixed with 2.5 ml of distilled water and 0.5 ml of FeCl_3_ (0.1 %). The absorbance of all solutions was measured at 700 nm and expressed as mg of ascorbic acid equivalent per g of powder (mg A/g powder) and mg of quercetin equivalent per g of powder (mg QE/g powder).

#### Total antioxidant activity

The evaluation of total antioxidant activity of the extracts was done by a phosphomolybdenum method Ravishankar *et al*. [37]. 0.3 ml of extract was combined with 3 ml reagent solution (0.6 M sulfuric acid, 28 mm sodium phosphate and 4 mM ammonium molybdate). The reaction mixture was capped and incubated at 95 °C for 90 min. After cooling to room temperature, the absorbance was measured at 695 nm against blank (methanol 0.3 ml). Ascorbic acid was taken as the standard.

### Anti-inflammatory activity

#### Albumin denaturation assay

A solution of 0.2 % *w/v* of Bovine serum albumin (BSA) was prepared in Tris buffer (pH 6.8). Both extract and standard drugs (diclofenac sodium) were diluted in concentrations: 500, 1000, 1500, 2000 and 2500 μg/ml). 5 ml of 0.2 % *w/v* BSA was transferred to tube containing 50 μg/mL of extract/standard. The control tube consists of 5 mL 0.2 % *w/v* BSA solution with 50 μl methanol. The samples was heated at 72 °C for 5 min and cooled at room temperature for 15 min [38, 39]. The optical density of the solution was read at 660 nm and percentage inhibition of precipitation (denaturation of proteins) was determined as compared to control using following formula: % Inhibition = (Abs control - Abs sample) /Abscontrol × 100.

#### Membrane stabilization assay

Human red blood cells (HRBC) membrane stabilization method was used to study the anti-inflammatory activity. Blood was collected from healthy volunteers who was not taken any analgesic medication for two weeks and mixed with equal volume of sterilized Alsever solution (2 % dextrose, 0.8 % sodium citrate, 0.5 % citric acid and 0.42 % sodium chloride in water). Blood was centrifuged at 3000 RPM for 15 min. Packed cells were washed with isosaline (0.85 %, pH 7.2) and a suspension was made with isosaline (10 %). Different concentrations of extract: 50, 100, 250, 500 and 1000 ug/ml were prepared in isosaline. The assay mixture contained 0.5 ml of HRBC suspension, phosphate buffer (0.15 M pH 7.2), 2 ml hyposaline (0.36 %) and 1 ml of various concentrations of extract and incubated at 37 °C for 30 min. Then, the mixture was centrifuged at 3000 RPM for 20 min. Diclofenac sodium was used as reference standard [40, 41]. The absorbance of supernatant solution was estimated using spectrophotometer at 560 nm.

$$ \begin{array}{l}\%\ \mathrm{Hemolysis}\ \mathrm{was}\ \mathrm{calculated}\ \mathrm{b}\mathrm{y}:\ \mathrm{O}\mathrm{D}\ \mathrm{of}\ \mathrm{test}/\mathrm{O}\mathrm{D}\ \mathrm{of}\ \mathrm{control} \times 100\hfill \\ {}\mathrm{Percentage}\ \mathrm{protection} = 100 - \mathrm{O}\mathrm{D}\ \mathrm{of}\ \mathrm{the}\ \mathrm{test}/\mathrm{O}\mathrm{D}\ \mathrm{of}\ \mathrm{control} \times 100\hfill \end{array} $$

### Antimicrobial activity

#### Procurement of microorganisms

The bacterial strains were obtained from Institute of Microbial Technology, Chandigarh. The bacterial species: gram-positive *Staphylococus aureus (S. aureus)* (MTCC 737), gram-negative *Pseudomonas aeruginosa* (*P. aeruginosa*) (MTCC 741) and *Escherichia coli* (*E. coli*) (MTCC 739), *Klebsiella pneumoniae* (*K. pneumoniae*) MTCC 1427), and yeast represented by *Candida albicans* (MTCC 3958) *Saccharomyces cerevisiae* (MTCC 827) were used for evaluating antimicrobial activity.

#### Determination of antibacterial and antifungal activities

##### Antibacterial activity

Muller Hinton agar plates with 4 % NaCl supplementation were prepared. Sterilized swabs were dipped in standardized bacterial suspension with an inoculum size of 1.5 × 10^8^ cfu/ml prepared above and excess culture was removed by turning the swab against the side of the tube. Inoculum was spread evenly over the entire surface of Muller Hinton Agar plates. These plates were allowed to dry for at least 15 min and then well (7 mm diameter) were made on petri dish using sterile cork borer. About 25 μl extracts were introduced into bore agar wells using a sterile dropping pipette. These plates were kept inside the refrigerator at 4 °C for 6 h to allow proper diffusion of extracts into the medium. The plates were then examined for antibacterial activities of extracts after 24 h of incubation at 37 °C [42, 43]. Antimicrobial activity was determined by measuring the diameter zone of inhibition in mm.

##### Antifungal activity

Sabouraud dextrose agar (SDA) plates were prepared and sterilized swabs were dipped in standardized fungal suspension with an inoculum size of 1.5 × 10^7^ cfu/ml prepared above and excess culture was removed by turning the swab against the side of the tube. Inoculum was spread evenly over the entire surface of SDA plates. These plates were allowed to dry for at least 15 min and then well (7 mm diameter) were made on petri dish using sterile cork borer. About 25 μl extracts were introduced into bore agar wells using a sterile dropping pipette. These plates were kept inside the refrigerator at 4 °C for 6 h to allow proper diffusion of extracts into the medium. The plates were then examined for antifungal activities of extracts after 72 h of incubation at 25 °C. The antimicrobial activity was determined by measuring the diameter zone of inhibition in mm [42, 43].

### Statistical analysis

Results were expressed as mean ± standard deviation (SD). Statistical analysis was performed by one-way ANOVA followed by Bonferroni’s multicomparison test as *post hoc*. The software GraphPad Prism (version 6.0) was used and a probability (*p*) value < 0.05 was considered to be statistically significant.

## Results

### Phytochemical screening of plant extracts

The qualitative phytochemical analysis of the three plant extracts revealed the presence of alkaloids, flavonoids, carbohydrate, glycosides, steroids, tannin and phenolics (terpenoids). The presence of these classes of constituents may signify the biological activity of the plants. The observations made during phytochemical analysis are shown in Table 1.

**Table 1:** Phytochemical screening of the plant extracts of three Pine species

Compound	Detection method	*P. roxburghii*	*P. wallichiana*	*P. gerardiana*
Alkaloids	Dragendroff test, Mayers test, Wagners test	+	+	+
Flavonoids	Ferric chloride test	+	+	+
Amino acids	Ninhydrine test	−	−	−
Carbohydrates	Mollisch test	+	+	+
Fats and oils	Sudan 3	+	+	+
Tannins	Gelatin test	+	+	+
Steroids	Salkowski test, sulfur powder test	+	+	+
Saponin Glycosides	Froth floatation test	+	+	+
Terpenoids	Lead acetate test	+	+	+
Phenolics	Salkowski’s test	+	+	+

(+) Positive, (−) = Negative

### Total phenolic, flavonoid and tannin content in plant extracts

In the present study, the phenolic content estimated in *P. roxburghii* was 246.66 ± 1.52 mg GAE/g, *P. wallichiana* 222.33 ± 1.15 mg GAE/g and *P. gerardiana* 248.66 ± 0.57 mg GAE/g. The total flavonoid content estimated in *P. roxburghii* was 597.14 ± 0.73 mg QR/g, *P. wallichiana* 476.55 ± 0.42 mg QR/g and *P. gerardiana* 535.23 ± 0.48 mg QR/g. The tannin content found in *P. roxburghii*, *P. wallichiana* and *P. gerardiana* extracts were 80.43 ± 1.3 mg QR/g, 72.34 ± 0.5 mg QR/g, and 68.41 ± 0.3 mg QR/g respectively (Table 2).

**Table 2:** Total antioxidant capacity, phenol content, flavonoid, β-carotene, lycopene and tannin contents in plant extracts

Plant	Polyphenol content (mg of GAE/g DW)	Flavonoid content (mg of QR/g DW)	Tannin content (mg of QR/g DW)	β- carotene (μg/mg)	Lycopene (μg/mg)	Total antioxidant capacity (mg of GAE/g DW)
*P. roxburghii*	246.66 ± 1.52	597.14 ± 0.73	80.43 ± 1.3	0.1034 ± 0.001	0.065 ± 0.003	221.33 ± 0.6
*P. wallchiana*	222.33 ± 1.15	476.55 ± 0.42	72.34 ± 0.5	0.1054 ± 0.001	0.070 ± 0.001	202.21 ± 1.12
*P. gerardiana*	248.66 ± 0.57	535.23 ± 0.48	68.41 ± 0.3	0.104 ± 0.001	0.076 ± 0.0004	215.03 ± 0.42

### Estimation of β-carotene and lycopene in plant extracts

The results obtained in the present study showed carotene levels in *P. roxburghii* 0.1034 ± 0.001; *P. wallichiana* 0.1054 ± 0.001 and in *P. gerardiana* 0.104 ± 0.001. The lycopene content obtained in present investigation were 0.065 ± 0.003 in *P.roxburghii*, 0.070 ± 0.001 in *P. wallichiana* and 0.076 ± 0.004 in *P. gerardiana* (Table 2). These findings are reported for the first time for Pinus plant species.

### Antioxidant activity of plant extracts

The diversity of nature and complexity of phytochemical compounds obtained from plant extracts affects the efficacy in various estimations. Hence, assessments involving various methods are reliable to estimate the effectiveness of substances. In the present study, five methods have been used to assess antioxidant activities of three plant extracts from Pinus plant species which are: DPPH radical scavenging assay, nitric oxide assay, reducing power assay, H_2_O_2_ scavenging assay and total antioxidant activity.

#### DPPH radical scavenging assay

**Fig. 1:**
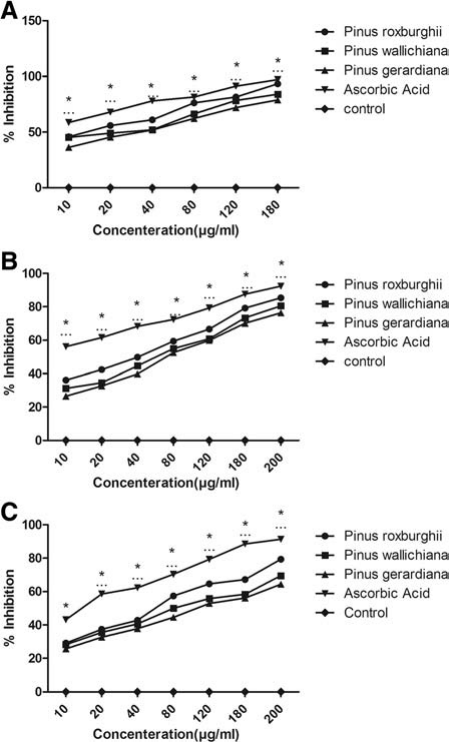
Antioxidant activities of the plant extracts estimated using: **a** DPPH radical scavenging activity, **b** Nitric oxide Radical scavenging activity, and **c** Hydrogen peroxide radical scavenging activity; * *p* < 0.05 vs control at respective concentration

The antioxidant activity of a compound is the amount needed to decrease the initial DPPH concentration by 50 % and expressed as IC_50_. DPPH radical scavenging activity of hydro-alcoholic extracts of *P. roxburghii*, *P. gerardiana* and *P. wallichiana* were compared with ascorbic acid (standard) and presented in Table 3. The results showed the degree of discoloration and indicate significant free radical scavenging activity in terms of IC_50_ values of *P. roxburghii* (97.54 ± 0.67 μg/ml), *P. wallichiana* (111.40 ± 0.78 μg/ml) and *P. gerardiana* (102.86 ± 1.2 μg/ml). The percentage inhibition was calculated and presented in Fig. 1a.

**Table 3:** Free radical scavenging activity of plant extracts

Plants	IC_50/DPPH_ (μg/ml)	IC_50_/H_2_O_2_ (μg/ml)	IC_50_/NO_2_ (μg/ml)
*P. roxburghii*	97.54 ± 0.67	86.90 ± 1.2	111.38 ± 1.8
*P. wallichiana*	111.40 ± 0.78	84.18 ± 0.67	98.5 ± 2.1
*P. gerardiana*	102.86 ± 1.2	81.83 ± 0.84	109.23 ± 0.65
Ascorbic acid	18 ± 2.1	16.72 ± 0.42	17.99 ± 0.34

#### Nitric oxide radical scavenging assay

Sodium nitroprusside generates NO as free radical in aqueous solution at physiological pH, which reacts with oxygen to form nitrites, oxides of nitrogen [44]. The formation and scavenging of NO by plant extracts were found to be comparable to standard drug and presented as IC_50_ values: 17.99 ± 0.34 μg/ml for ascorbic acid, 111.38 ± 1.8 μg/ml for *P.roxburghii*, 98.5 ± 2.1 μg/ml for *P. wallichiana* and 109.23 ± 0.65 μg/ml for *P. gerardiana* in Table 3 and percentage inhibition graph is plotted in Fig. 1b.

#### Hydrogen peroxide radical scavenging assay

The potential of plant extracts in scavenging hydrogen peroxide was attributed to the presence of phenols and tannins which could donate electrons, thereby neutralizing it into water [45]. The results showed that there is a scaling increase in the scavenging of H_2_O_2_ due to different concentration of Pinus extracts. IC_50_ values were calculated and presented in Table 3 and percentage inhibition graph is shown in Fig. 1c. The IC_50_ values were found to be as: ascorbic acid 16.72 ± 0.42 μg/ml, *P. roxburghii* 86.9 ± 1.2 μg/ml, *P. wallichiana* 84.18 ± 0.67 μg/ml and *P. gerardiana* 81.83 ± 0.84 μg/ml. *P. roxburghii* exhibited promising H_2_O_2_ scavenging activity.

#### Reducing power assay

The reducing capabilities of all three plant extracts showed significant electron donating property which was found to be comparable with ascorbic acid (standard). The findings of reducing power assay of all plant extracts were presented in Table 4.

**Table 4:** Reducing power assay of plant extracts

S.No.	Conc (μg/ml)	*P. roxburghii* absorbance (700 nm)	*P. wallichiana* absorbance (700 nm)	*P. gerardiana* absorbance (700 nm)	Ascorbic acid absorbance (700 nm)
1.	10	0.304 ± 0.23	0.237 ± 0.64	0.325 ± 0.12	0.377 ± 0.19
2.	20	0.454 ± 0.34	0.423 ± 0.98	0.463 ± 0.54	0.491 ± 0.78
3.	40	0.569 ± 0.67	0.489 ± 1.17	0.511 ± 0.67	0.545 ± 0.45
4.	80	0.642 ± 0.78	0.507 ± 0.78	0.626 ± 0.23	0.679 ± 0.34
5.	120	0.762 ± 0.32	0.593 ± 0.54	0.652 ± 0.56	0.753 ± 0.41
7.	180	0.824 ± 1.12	0.693 ± 0.32	0.792 ± 0.32	0.782 ± 0.63
8.	200	0.842 ± 0.78	0.753 ± 0.11	0.812 ± 0.21	0.854 ± 0.43

#### Total antioxidant activity

The results obtained using phosphomolybdate methods for total antioxidant activities of all plant extracts were found to be *P. roxburghii* 221.33 ± 0.6 mg GAE/g, *P. wallichiana* 202.21 ± 1.12 mg GAE/g and *P. gerardiana* 215.03 ± 0.42 mg GAE/g (Table 2). Methanolic extract of leaves and fruits of various Pinus species from Iran, including *P. wallichiana* had shown significant antioxidant activity when compared to alpha-tocopherol [46]. In another report, several methods have been used to assess total anti-oxidant capacity of *P. gerardiana* nuts, also provided an insight to the solubility of antioxidant compounds in different types of solvents [24].

### Anti-inflammatory activity of plant extracts

The results of *the in-vitro* assessment of anti-inflammatory activity of plant extract is described as:

#### Albumin denaturation assay

Results from the present study showed that there is a scaling increase in albumin denaturation assay in Pinus species, thereby can be used as potential anti-inflammatory agents. Further *in-vivo* study is required to elucidate its exact mechanism of action. *P. roxburghii* exhibited highest inhibition against albumin denaturation 86.54 ± 1.85 whereas *P. gerardiana was* having 82.03 ± 2.67 Table 5(A).

**Table 5:** Anti-inflammatory activities of the plant extracts estimated using (A) Albumin denaturation assay, and (B) HRBC membrane stabilization assay. Each value represents the mean of three experiments and standard deviation of measurement

(A)					
Conc (μg/ml)	*Pinus roxburghii* (% inhibition)	*Pinus wallichiana* (% inhibition)	*Pinus gerardiana * (% inhibition)	Diclofenac sodium (% inhibition)	Control (% inhibition)
500	26.680 ± 2.48	21.080 ± 1.78	24.080 ± 1.12	58.030 ± 2.69	0.00 ± 0.00
1000	39.120 ± 1.13	34.820 ± 2.94	32.040 ± 2.19	68.050 ± 1.03	0.00 ± 0.00
1500	54.180 ± 3.74	46.630 ± 1.23	49.820 ± 1.54	76.040 ± 3.12	0.00 ± 0.00
2000	69.430 ± 1.13	64.080 ± 3.19	63.030 ± 1.23	85.030 ± 1.21	0.00 ± 0.00
2500	86.540 ± 1.85	76.540 ± 2.45	82.030 ± 2.67	92.040 ± 1.23	0.00 ± 0.00
(B)					
Conc (μg/ml)	*Pinus roxburghii* (% protection)	*Pinus wallichiana* (% protection)	*Pinus gerardiana* (% protection)	Diclofenac sodium (% protection)	Control (% protection)
500	32.120 ± 1.32	27.540 ± 2.01	30.120 ± 2.18	61.030 ± 3.69	0.00 ± 0.00
1000	41.720 ± 2.16	35.920 ± 1.98	42.940 ± 1.43	70.050 ± 2.03	0.00 ± 0.00
1500	58.480 ± 2.54	46.130 ± 3.23	52.620 ± 3.14	79.041 ± 3.12	0.00 ± 0.00
2000	72.540 ± 3.19	67.840 ± 2.09	71.830 ± 1.12	87.030 ± 3.21	0.00 ± 0.00
2500	89.920 ± 2.64	81.240 ± 2.95	85.230 ± 2.47	94.840 ± 2.73	0.00 ± 0.00

#### HRBC membrane stabilization assay

The biological potential of plant extracts was studied for their ability to stabilize human RBC membrane lyses in hypotonic saline. The results obtained were also compared with standard anti-inflammatory agent diclofenac sodium and % protection showed by *P. roxburghii* at highest concentration was 89.92 ± 2.64; *P. wallichiana was* 81.24 ± 2.95; *P. gerardiana* was 85.23 ± 2.47 when compared with diclofenac (94.84 ± 2.73) Table 5(B).

### Antimicrobial activity

**Fig. 2:**
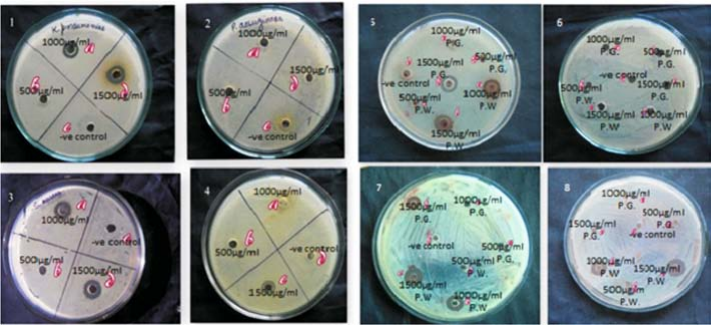
Bacterial inhibition zone using plant extracts: *P.roxburghii* extract: (1) *K. pueumoniae,* (2) *P aeruriginosa,* (3) *S. aureus,* (4) *E. coli*; *P. wallichiana* and *P. gerardiana* extracts: (5) *K. pueumoniae,* (6) *E. coli,* (7) *P. aeruriginosa,* (8) *S. aureus*

The antibacterial activity of plants extracts against Gram positive (*S. aureus*) and Gram negative (*E. coli, P. aeruginosa* and *K. pneumoniae*) bacteria were assessed by agar well diffusion plate method by estimating the diameter of zone of inhibition. Hydro-alcoholic extract of *P.wallichiana* possessed potent antibacterial activity amongst three plant extracts. The diameter of zone of inhibition by all plant extracts is presented in Table 6 and zone of inhibition are shown in Fig. 2.

**Table 6:** Antibacterial activity of plant extracts (inhibition zone)

Sample	Concentration (μg/ml)	Inhibition zone (mm)			
		*P. aeruginosa*	*S. aureus*	*E. coli*	*K. pneumonia*
*P. roxburghii*	500	–	–	–	–
	1000	–	10.2 ± 0.5	–	10.95 ± 0.5
	1500	–	12.1 ± 0.5	–	13.2 ± 0.5
*P.wallichiana*	500	–	–	–	–
	1000	11.25 ± 0.5	11.93 ± 0.5	–	10.12 ± 0.5
	1500	14.1 ± 0.5	14.21 ± 0.5	–	12.3 ± 0.5
*P. gerardiana*	500	–	–	–	–
	1000	–	–	–	–
	1500	10.05 ± 0.5	–	–	–

### Antifungal activity

**Fig. 3:**
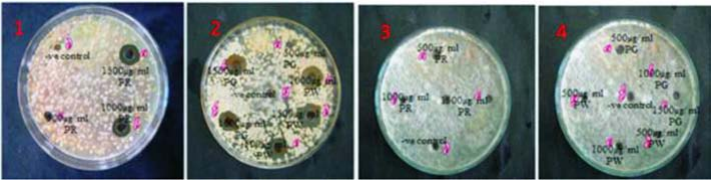
Fungal inhibition zone using plant extracts: *P.roxburghii* extract (1) and *P.wallichiana* and *P. gerardiana* (2) against *Candida albicans*; *P.roxburghii* (3) and *P. wallichiana* and *P.gerardiana* (4) against *Saccharomyces cereveseae*

It would definitely be a better alternative to search of natural fungicide from plant source instead of hazardous chemicals. The extracts of Pinus plants used showed prominent antifungal activity against *Candida albicans* but they showed no activity against *Saccharomyces cerevisiae*. The diameter of zone of inhibition with use of all plant extracts along with standard are illustrated in Table 7 and zone of inhibitions are shown in Fig. 3. Thus, the use of crude bark extracts of Pinus plant species in treatment of pathogenic diseases associated with the pathogens can be scientifically supported by the findings from the present study.

**Table 7:** Antifungal activity of plant extracts (inhibition zone)

Sample	Concentration (μg/ml)	Inhibition zone (mm)	
		*Candida albican*	*Saccharomyces cereveseae*
*P. roxburghii*	500	–	–
	1000	13.1 ± 0.5	–
	1500	15.3 ± 0.5	–
*P. wallichiana*	500	14.05 ± 0.5	–
	1000	17.23 ± 0.5	–
	1500	18.93 ± 0.5	–
*P. gerardiana*	500	–	–
	1000	12.15 ± 0.5	–
	1500	15.01 ± 0.5	–

## Discussion

The present study elaborates that medicinal plants are laoded with diverse pharmacological actions and the findings of present study are the contribution to the valorisation of three Pinus species from Himachal Pradesh, two of which have never been explored scientifically. Free radicals are constantly generated in the living systems, and when in excess can cause extensive damage to the tissues and biomolecules leading to pathological condition like inflammation, cell death and organ failure. The scavenging of free radicals using antioxidants may offer resistance to oxidative stress and cell damage [47–49]^.^ DPPH radical method is considered to be a role model for assessment of anti-oxidant action. The test is based upon the fact DPPH (deep violet colour) is stable free radical, when reacted with anti-oxidants converts to yellow coloured compound: di phenyl hydrazine [44, 50, 51]. DPPH radical scavenging activity of hydro alcoholic extract of *P. roxburghii*, *P. gerardiana* and *P. wallichiana* was compared with ascorbic acid that serves as a positive control. Nitric oxide is an essential bio regulatory radical produced in mammalian cells, and even the potent pleiotropic mediator of physiological processes, involved in the regulation of various physiological reactions, including oxidative & nitrosative injuries, release of pro-inflammatory mediators like TNF-α, interleukins and activation of caspases resulting fatal conditions. Sodium nitroprusside generates NO free radical in aqueous solution at physiological pH, which reacts with oxygen to form nitrites oxides of nitrogen [45]. The scavenging activity of plant extract against nitric oxide formation was compared with standard drug. Hydrogen peroxide, although not a radical, upon catalytic conversion it produces deleterious hydroxyl radicals. Scavenging activity of Pinus extracts may attribute the presence of phenolic group, which can donate electrons to hydrogen peroxidase, thus neutralizing it to water [52]. The comparison of H_2_O_2_ radical scavenging activity was compared with ascorbic acid. Evaluation of antioxidant activity of molecule can be made by monitoring their ability to reduce Fe3^+^ iron ion intoFe2^+^. If the fenton reaction undergoes, it may result in the formation of highly reactive hydroxyl radicals and this contributes to oxidative stress [53, 54]. This Fe2^+^ can be monitored by measuring the formation of Perl’s Prussian blue at 700 nm. Iron is an important mineral but in excess it may cause cellular injury. The reducing capabilities of *P. roxburghii*, *P. wallichiana*, and *P. gerardiana* were compared with ascorbic acid. Inflammation is a complex biological response, to remove injurious stimuli as well as initiate the healing process. It is a biological defensive response for the management of pro-inflammatory conditions. The medicinal plants and the constituents seem to be viable and logical alternative to treat inflammatory pathological state. A simple and viable protein denaturation and HRBC membrane stabilization methods are used to study *in-vitro* anti-inflammatory activity of plant extracts [55]. Pinus plant extracts inhibited the hypotonicity induced lysis of erythrocyte membrane, exhibited membrane stabilization effect to lysosymal membrane and thus showed a significant anti-inflammatory effect.

In the last three decades pharmaceutical industries are involved in the search for development of newer antibiotics have been increased and become a global concern [56]. Thus infections with the microbes have always been considered with high mortality and morbidity especially with immune compromised patients. The search for new chemotherapeutic alternatives from traditional medicine lead to a great success to eliminate the infections caused by drug-resistant microbes and to reduce the harm caused by antibiotics. All the plant extracts from each Pinus species have shown comparable antimicrobial and antifungal activities, as presented by zone of inhibition against bacteria.

Hence the present investigation suggests that Pinus plant extracts of *P. roxburghii*, *P. wallichiana* and *P. gerardiana* and their constituents are capable of scavenging free radicals, decreasing pro-inflammatory mediators and providing protection against microbial infections, and these biological properties may be attributed to the potential of different constituents like phenolics, terpenes, flavonoids etc. *P. roxburghii* is only reported to have anti-oxidant and anti-inflammatory effect; hence, the findings of the present study may extend to provide scientific rationale for the therapeutic uses of Pinus species especially *P. wallichiana* and *P. gerardiana*, for the first time. These findings are preliminary and perhaps the basis for evaluation of *in-vivo* pharmacological potential of extracts and fractions of the three pinus species for disorders like neurodegeneration, osteoporosis, inflammation, which are also being conducted in our laboratory.

## Conclusion

The scientific data available for the biological potential of pinus plant species and their constituents is found to be scanty and also do not satisfy the basis of their age old folklore and local uses. The findings from present investigation have come up with a concrete view of the abilities of pinus plant components like phenolics, flavonoids, tannins and other constituents to treat oxidative, inflammatory and microbial responses *in-vitro* for the first time. Conclusively, the active phytoconstituents from Pinus plant species which abundantly covers the Indian Himalayan region, are of great research interest to develop novel therapeutics for the welfare of mankind.
